# Fungous Origin of Scarlet Fever

**Published:** 1869-08

**Authors:** 


### Fungous Origin of Scarlet Fever.

Hallier, who has distinguished himself so greatly by his investigation of the cholera fungus, has discovered something similar in scarlet fever blood. He says he has never seen such an immense number of micrococci in the blood of any other infectious disease. These are at first as small as the finest pin-point, or the most minute granular matter. They are present in far greater numbers than the blood globule themselves; both swimming free in the serum and accumulated in granular masses and groups. They both accumulate on and penetrate into the blood globules. The white corpuscles as well as the red globules are supplied with them, almost without exception. Just as there is a great resemblance between the seeds, roots, stems and leaves of plants, so do the microscopic germs develop themselves into sprouts and shoots which resemble those of many other microscopic plants. But Hallier has discovered three immature and three mature forms of spores, which he considers characteristic of scarlet fever poisoning, and names them TILLETIA SCARLATINOSA. J. C. P.

*** Prof. Hallier, in his researches in Microscopic botany, has cultivated many fungi up to the reproductive or seed bearing stage.

In this mature (coniothecal) form, characteristic specific differences are noticeable between the different plants; but during the earlier stages of developement it is almost impossible to distinguish between them. Nearly all begin as micrococci, and pass through the various forms of torula and penicillium, before showing marked individual characteristics.—*Medical Gazette.*

———:o:———

At a recent meeting of the French Academy of Medicine, a paper by Dr. Shrimpton was read, calling attention to the great modifications of the hospital system taking place in England. He pointed out that the large hospitals in London and other towns were likely to disappear gradually, in consequence of the recognized superiority of cottage hospitals scattered all over the country. To show conclusively the superiority of these cottage hospitals, Dr. Shrimpton adduced some very remarkable facts vouched for by Sir James Simpson, from which it appears that the mortality after surgical operations is very much less in these small hospitals—where the patients are free from the obnoxious effects of overcrowdings and enjoy the advantage of pure country air—than in the large hospitals of London, Edinburg, and Glasgow. These figures speak for themselves—thus out of 2,089 amputations in the large town hospitals, 825 proved fatal, whilst in the country, out of 2,098 amputations, a fatal result ensued only in 226 cases. This communication produced a great impression on the Academy of Medicine, and well it might, for the mortality in the French hospitals, especially through the overcrowding and bad ventilation of the wards, is something perfectly fearful. Placed almost invariably in the most unhealthy parts of Paris, the hospitals are so crowded that the patients have not their proper proportion of air. The great bulk of French medical men look upon these huge caravanserais of sickness more as places of study for the profession than places of cure for the patient. The consequence is, that the lower classes in Paris have as great a dread of the hospital as the lower classes with us of the workhouse, and these establishments professedly raised for the relief of the suffering poor, are chiefly useful as experimental schools of medicine. Instead of being what they are intended to be, *sanatoria*, they are merely *mortuaria*—huge museums of disease. Dr. Shrimpton has done good service in drawing the attention of his French *confreres* to cottage hospitals, and their obvious superiority, and it is to be hoped that some of the French medical reformers will take the matter vigorously up.—*Medical Gazette.*

